# HISTAI: a valuable dataset with a valuable lesson

**DOI:** 10.1002/2056-4538.70089

**Published:** 2026-04-15

**Authors:** Katherine J Hewitt, Nic G Reitsam, Sebastian Foersch, Ramona Erber, Qinghe Zeng, Jakob Nikolas Kather

**Affiliations:** ^1^ Else Kroener Fresenius Center for Digital Health, Faculty of Medicine and University Hospital Carl Gustav Carus TUD Dresden University of Technology Dresden Germany; ^2^ Pathology, Faculty of Medicine University of Augsburg Augsburg Germany; ^3^ Bavarian Center for Cancer Research (BZKF) Augsburg Germany; ^4^ Institute of Pathology University Medical Center Mainz Mainz Germany; ^5^ Institut für Pathologie Universität Regensburg Regensburg Germany; ^6^ Pathologisches Institut Universitätsklinikum Erlangen Erlangen Germany; ^7^ Department of Medicine I, Faculty of Medicine and University Hospital Carl Gustav Carus TUD Dresden University of Technology Dresden Germany; ^8^ Medical Oncology, National Center for Tumor Diseases (NCT) University Hospital Heidelberg Heidelberg Germany; ^9^ Pathology & Data Analytics, Leeds Institute of Medical Research at St James's University of Leeds Leeds UK

**Keywords:** computational pathology, open‐access datasets, HISTAI, ground truth validation, data quality and reproducibility, diagnostic concordance

## Abstract

The application of artificial intelligence in computational pathology depends on both robust algorithms and high‐quality, clinically reliable data. Progress in this field has been limited by the scarcity of large, diverse, and well‐validated whole slide image (WSI) datasets. To address this gap, HISTAI introduced an open‐source resource comprising over 112,000 WSIs across multiple organ systems with associated clinical metadata. Here, we present a pathologist‐led evaluation of label accuracy, metadata completeness, and dataset composition across 328 selected cases from this resource. Although HISTAI reports 47,279 cases, we identified only 44,564 unique cases after accounting for missing entries and duplicate records. Basic demographic information, including age and sex, was available for only 55% of cases. Dataset composition was uneven, with dermatopathology accounting for 47.1% of cases and gastrointestinal pathology for 24.0%; however, primary specialty was explicitly reported for only 39.6% of cases, obscuring this imbalance within the provided metadata. Notably, clinical ground truth is recorded in the Conclusion column. Concordance between the dataset's Conclusion and Diagnosis fields was observed in only 20.7% of cases, while 27.1% contained conflicting diagnoses. In a focused review of 198 cases, 30.3% were found to contain unclear or ambiguous diagnostic conclusions, including eight cases in which the diagnosis was incorrect. Assessment of molecular annotation revealed that only 18.9% of analyzed lung and colorectal cancer cases included molecular information. Furthermore, among adult‐type diffuse gliomas, none of the 55 cases met current World Health Organisation Classification of Tumors of the Central Nervous System 5th Edition (WHO CNS5) diagnostic criteria, with *IDH* mutation status reported in only 15 cases. Together, these findings highlight substantial ambiguities in ground‐truth labeling, incomplete molecular annotation, and limited documentation of dataset provenance and ethical oversight. While HISTAI represents a valuable open‐source resource, its effective and responsible use requires careful clinical validation and close collaboration between computational researchers and pathologists.

## Introduction

Artificial intelligence in computational pathology is defined by two key factors: algorithms and data. Despite advances in computational methods in recent years, a major limitation remains the scarcity of large, diverse, and openly available datasets. Without access to high‐quality, clinically validated digitized images, even the most advanced algorithms risk producing misleading or inaccurate outputs.

The cancer genome atlas [1] (TCGA) continues to be the most widely used and diverse data resource. TCGA provides whole slide images (WSIs) together with a wealth of multi‐omic and clinical data, yet has shortcomings in terms of consistency and breadth [2, 3, 4]. Other public datasets, such as CPTAC [5], SurGen [6] and NCT100k [7] have contributed important benchmarks but are restricted either by scale, typically containing only hundreds to a few thousand images, or by focusing on a single organ system.

Compounding this issue, clinical metadata in many datasets is sparsely annotated or outdated, reducing their clinical utility. The scarcity of publicly available, clinically annotated WSI cohorts spanning the full diagnostic spectrum with rigorous ground truth remains a central barrier, restricting realistic training and undermining credible external validation needed for clinical implementation.

To address this unmet need, the HISTAI dataset was first introduced in a paper submitted to arXiv on 17 May 2025 [8]. The authors present HISTAI as a large, open‐source WSI dataset designed to overcome the recognized limitations of existing public resources, namely restricted scale, limited tissue diversity, and sparse clinical annotations. As of February 2026, the dataset contained more than 112,000 WSIs.

The dataset is organized into nine directories, entitled Breast, Colorectal‐B1, Colorectal‐B2, Gastrointestinal, Hematologic, Skin‐B1, Skin‐B2, Thorax, and Mixed. These directories are not further subcategorized and the largest directory, the Mixed cohort, contains 52,691 WSIs representing 21,137 cases.

The vast majority of slides (*n* = 92,536) are stained with hematoxylin and eosin (H&E), with approximately 110,338 slides scanned at ×20 magnification and 2,463 slides at ×40. In terms of other staining protocols, 16,920 slides are immunohistochemistry, while 3,345 employ alternative techniques. Each case is supplemented with associated clinical metadata, including clinical diagnostic information, a pathological conclusion, microscopic description, and basic demographic information such as patient age and gender. The dataset is openly accessible (following registration and granting access) via Hugging Face [9], making it freely available to the wider research community.

However, HISTAI's value to computational pathology ultimately hinges on the fidelity of its clinical ground truth and metadata; accordingly, we conducted a pathologist‐led appraisal of label quality and image‐report concordance on a subset of cases.

## Materials and methods

We accessed the entire HISTAI dataset via Hugging Face [9] and performed a data analysis of the clinical metadata, assessing the data composition, distribution, and completeness of the clinical data. We then reviewed 328 unique cases composed of 100 cases randomly selected from the Thorax (*n* = 50) and Colorectal B1 (*n* = 50) cohorts, 98 cases randomly sampled across all datasets, and 130 Neuropathology cases, identified using a key word search.

In this dataset, the ground truth diagnosis is recorded in the Conclusion column rather than the Diagnosis column. Concordance between these fields was assessed to quantify the potential impact of mistakenly using the Diagnosis column as ground truth. The diagnostic concordance was rated as either Concordant, when the diagnosis in both columns was aligned, Discordant, when the diagnoses were different, or Not Clinically Specific, when the diagnoses fell broadly into the same diagnostic category but were not strictly concordant. Diversity was assessed by classifying cases according to their ground truth as either Malignant, Benign Tumor, or Other.

The images for the 98 randomly sampled cases and the 100 Thorax and Colorectal cases were reviewed to assess if the diagnosis given in the Conclusion column aligned with the appearance on H&E and the microscopic description. Furthermore, within the Neuropathology, Thorax, and Colorectal cases, we identified whether relevant molecular information was available within the clinical metadata.

## Results

We first reviewed the clinical metadata for the entire dataset. Although HISTAI reports 47,279 cases in the dataset [9], the metadata spreadsheet available via HISTAI's Hugging Face repository contained only 46,128 case entries. Of these, 1,549 were exact duplicates, resulting in an effective total of 44,564 unique cases. Basic demographic information is missing for around 45% of cases and among those with reported sex, there was a predominance of female patients.

**Figure 1:**
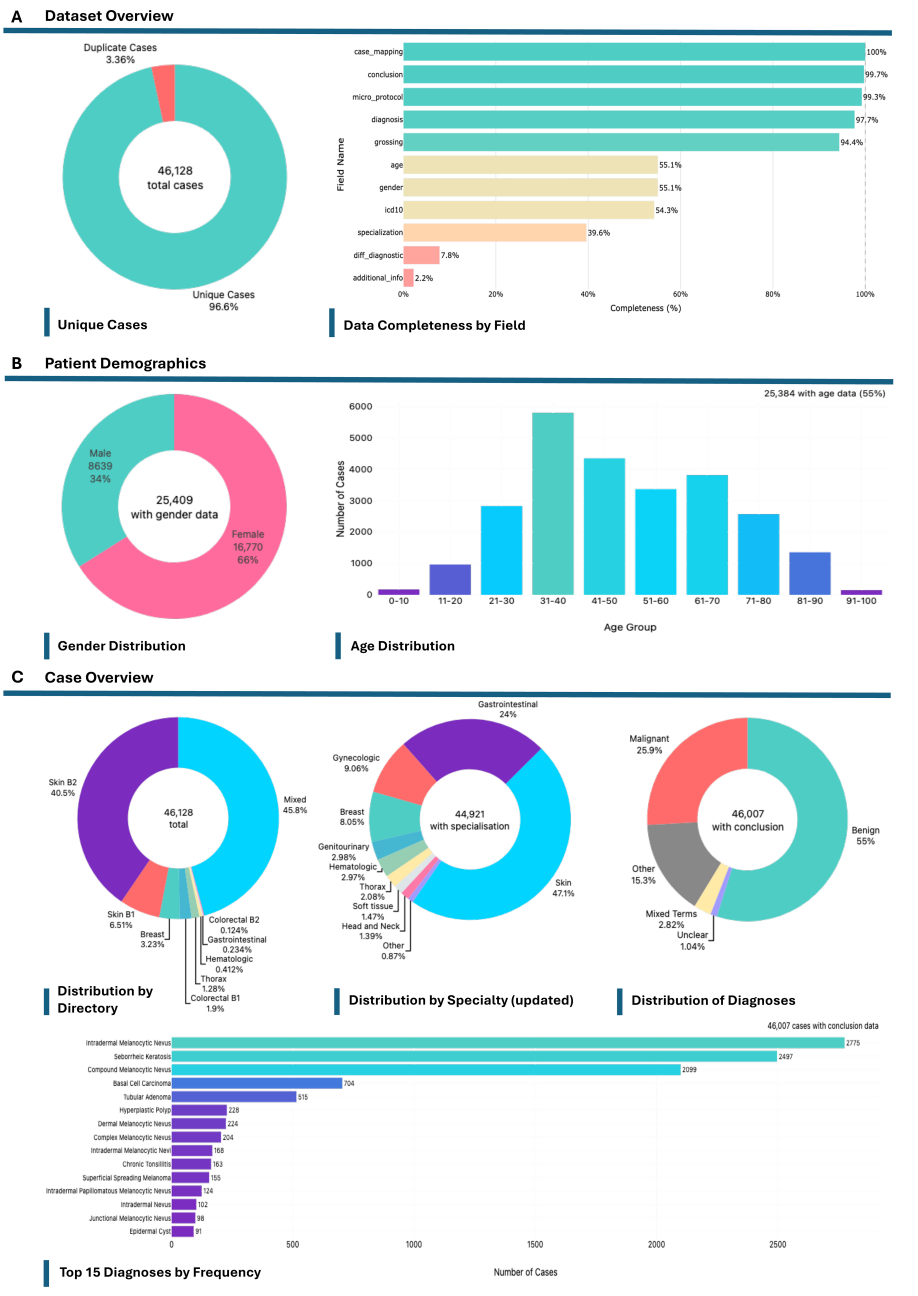
Dataset composition and metadata quality. (A) Although HISTAI reports a total of 47,279 cases, we found only 46,128 entries in the metadata spreadsheet and 1,549 of these were exact duplicates. (B) Basic patient demographics (age and sex) were available for only 55% of cases and showed a strong skew towards female patients. (C) Overview of diagnostic specialties represented in the dataset. The majority of cases (86.3%) were held in the Mixed and Skin B2 datasets. Specialty information was originally reported for only 39.6% cases; however, by extracting information from the Micro Protocol and Grossing columns, a specialty could be assigned to 97.4% of cases. Dermatology and Gastrointestinal cases together comprise 71.1% of the dataset, with all other specialties accounting for <10% of cases. This distribution is also reflected in the 15 most frequent diagnoses. Ground truth diagnoses are held in the Conclusion column, with 55.0% of the cases classified as benign and 25.9% of cases as malignant. Approximately 1,776 cases used unclear or ambiguous terminology such as ‘undetermined’, ‘suspicious’, or ‘cannot be excluded’ in their conclusion.

The largest proportion of cases (45.8%) were held in the Mixed directory; however, Specialization was reported for only 39.6% cases. By incorporating information from the Micro protocol and Grossing columns, Specialization coverage increased to 97.4%. Based on the updated Specialization data, 47.1% of cases were classified as dermatology, 24.0% as gastrointestinal, and the remaining 28.9% comprised all other specialties. Ground truth diagnosis extracted from the Conclusion column indicated that approximately 55.0% of cases were benign, 25.9% were malignant, and 1,776 cases used unclear or ambiguous diagnostic terminology. A summary of these findings can be found in Figure 1.

We next assessed diagnostic diversity across the 328 selected cases. The majority of cases were malignant (*n* = 131, 39.9%) and encompassed a wide variety of tumor types. For example, the Thorax cases included different primary lung malignancies (squamous cell carcinoma, adenocarcinoma, neuroendocrine neoplasms), lymph node specimens containing lymphoma or metastatic lung carcinoma, and a liver specimen showing metastasis from a lung primary. Benign entities accounted for 34.5% (*n* = 113). Non‐tumor diagnoses comprised 12.2% of cases (*n* = 40). In 53 cases (16.2%), a definitive diagnosis could not be immediately established. This was due to the presence of multiple diagnoses without clear correlation to the associated case image(s) (*n* = 27), molecular report content in place of the diagnosis (*n* = 20), or conclusions indicating that further analysis was required to confirm the diagnosis (*n* = 6).

Concordance between the diagnosis given in the Conclusion and Diagnosis columns was assessed for all 328 cases; the diagnosis was consistent in only 20.7% of cases (*n* = 68). In 52.3% of cases (*n* = 172), the Diagnosis was not strictly incorrect but was not clinically specific (e.g., the diagnosis was given simply as ‘Lung Cancer’). The remaining cases (*n* = 89, 27.1%) had differing diagnoses in the Conclusion and Diagnosis columns.

**Figure 2:**
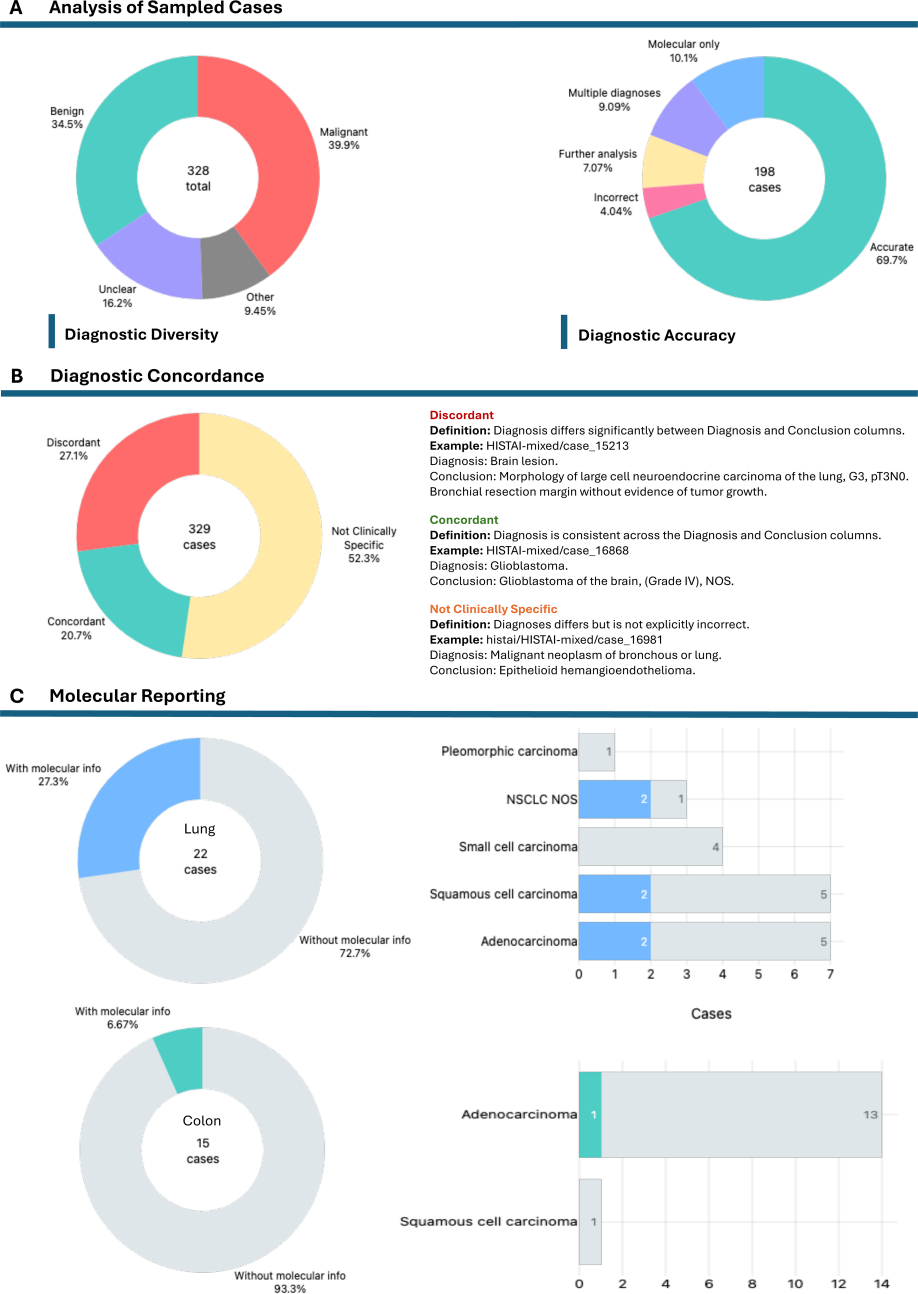
Diagnostic diversity, accuracy, and molecular reporting in sampled cases. (A) Diagnostic diversity across the 328 selected cases. Most cases were malignant, followed by benign entities. In 53 cases (16.2%), the diagnosis was unclear. A subset of cases (*n* = 198) were reviewed for diagnostic accuracy relative to the H&E slides. While most diagnoses were concordant with the histopathology, inaccuracies included molecular report content listed in place of a diagnosis (*n* = 20, 10.1%), multiple diagnoses without image correlation (*n* = 18, 9.1%), conclusions requiring further analysis (*n* = 14, 7.1%), and diagnoses inconsistent with the pathology shown (*n* = 8, 4.0%). (B) Diagnostic concordance between the Diagnosis and Conclusion columns. Only 20.7% of cases were fully concordant, 27.1% were discordant, and the remaining cases showed partial or nonspecific agreement. (C) Molecular reporting in the thoracic and colorectal cohorts. Among primary lung malignancies (*n* = 22), molecular information was reported in six cases (27.3%). Among primary colorectal malignancies (*n* = 15), molecular information was reported in one case (2.0%).

**Figure 3:**
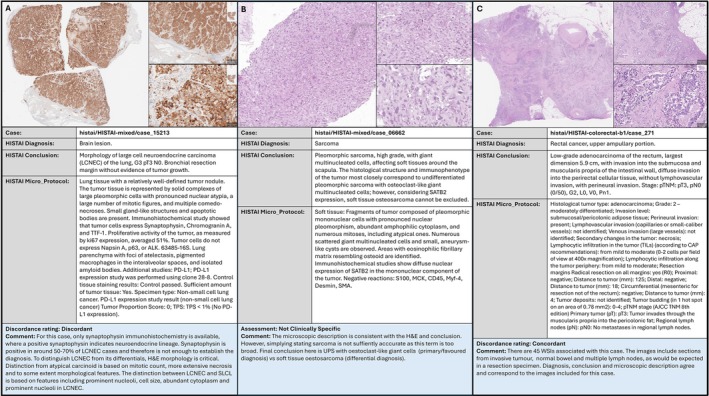
Example cases for each discordance category. This figure presents an example case for each of our three discordance categories, labeled as (A), (B), and (C). Cases were classified as Discordant when the diagnosis listed in the Diagnosis column and Conclusion column significantly differed. Cases were classified as Concordant when the diagnosis listed in both columns were consistent. However, in the majority of cases, the diagnosis in the Diagnosis column was generic, for example, Lung Cancer, while the Conclusion contained the histopathological subtype, for example, Epithelioid Hemangioendothelioma. These cases were classified as Not Clinically Specific. Each example includes one large, low‐resolution image and two higher resolution images of the case histology. Below the histology images are the Conclusion, Diagnosis, and Microscopic description from corresponding clinical metadata in the HISTAI dataset. Our discordance rating with an evaluation of the histology and clinical data are discussed in the blue box.

H&E slides for the Thorax, Colorectal, and randomly sampled cases (*n* = 198) were reviewed to assess accuracy between the histopathology, the microscopic description, and the diagnosis given in the Conclusion column. The majority of cases showed diagnoses that accurately reflected the H&E findings, with only minor discrepancies likely attributable to normal interobserver clinical judgement (69.7%, *n* = 138). However, in 60 cases [30.3%, bootstrap 95% CI (24.2%, 36.9%)] the Conclusion column did not contain an accurate diagnosis. Among these, 20 cases (10.1%) had conclusions that reported molecular findings without a corresponding diagnosis, 18 (9.09%) cases listed multiple diagnoses, and 14 cases (7.07%) indicated that further analysis was required to establish a definitive diagnosis. In the remaining eight cases (4.04%) the diagnosis given in the Conclusion column did not match the pathology shown in the case image. A summary of the Concordance and Diagnostic Accuracy results can be found in Figure 2. Figure 3 gives case examples for each concordance category.

Lastly, we assessed whether the diagnoses for adult‐type diffuse glioma (ATDG) cases met the current World Health Organisation Classification of Tumors of the Central Nervous System 5th Edition (WHO CNS5) diagnostic standards [10], and whether molecular data was included for the primary carcinoma cases included in the Thorax and Colorectal cases. The Thorax cases included 22 primary lung malignancies of which 6 (27.3%) included molecular information. The Colorectal cases included 15 primary malignancies (30.0%) of which 1 case (2.0%) included molecular information. Of the 130 cases that were identified as neuropathology, 55 cases were ATDG. None of these cases met, or could be updated to meet, current diagnostic standards using information provided within the dataset, and only 15 cases (27.3%) provided *IDH* mutation status information. Figure 2C provides an overview of the molecular reporting for the Thorax and Colorectal cases. An overview of the CNS WHO5 diagnostic criteria and a Consort chart for updating the diagnoses can be found in supplementary material, Table S1 and Figure S1.

## Discussion

While HISTAI offers an impressive volume and variety of data, it lacks a standardized organizational structure that facilitates use. Entities within the nine directories vary widely in etiology and are not strictly limited to the anatomical region or organ of the directory name, as one might reasonably expect. Datasets typically focus on one diagnostic category, such as a single primary malignancy, and while some public datasets contain multiple disease categories, these are typically stratified by diagnostic class through directory structure or metadata labeling [9]. Although this obstacle is by no means insurmountable, the issue is further compounded by the ground truth being stored in the Conclusion column, rather than the Diagnosis column. This detail is disclosed only in the Hugging Face metadata [11] and not mentioned either in the accompanying paper [8] or on the HISTAI official website [12].

It is our suspicion that the Diagnosis column contains the presumptive diagnosis provided by the referring clinicians to the pathologist and the Conclusion column contains the final pathological diagnosis. For clinicians and pathologists, this mismatch may be less misleading as they are used to this in their clinical workflow. However, for computational researchers who may be unfamiliar with clinical workflows, this nomenclature is highly ambiguous and risks misinterpretation of the ground truth. Specifically, if researchers were to mistakenly treat the ‘Diagnosis’ column as the ground truth, only about 20.7% of their labels would be clinically accurate, according to our estimation.

Beyond the unusual organization of the clinical data, closer inspection reveals concerns about the reliability of its clinical labels. Of the cases we examined, the ground truth was absent, unclear or incorrect in 30.3% [bootstrap CI (24.2%, 36.9%)]. Label inaccuracies, or noise, are present at some level in all datasets, and are known to introduce bias, limit achievable model performance, and reduce the reliability and reproducibility of downstream results [13, 14]. However, if this figure is representative for the entire dataset, this represents a significant issue for researchers using the data.

Additionally, gaps remain in the metadata from a clinical perspective. Genomic data is increasingly important, particularly in cancer diagnostics [15], and in some cases necessary for diagnosis [10]. Our analysis found that only 18.9% of the malignant lung and colorectal cases included molecular information. Additionally, *IDH* molecular alteration status has been an essential diagnostic criterion for what are now referred to as ATDGs since 2016, but only 27.3% of ATDG cases included such information. Furthermore, methylation profiling is not reflected in the dataset. Methylation profiling is of increasing importance, not only in brain tumors but also in other tumor entities, such as sarcomas [16, 17].

Significant concerns lie in the sparse information provided around data collection methods, sources, and the extent of clinician oversight. There is little transparency regarding the organizational approach to collection of the dataset, how cases were selected, or whether the slides included are representative of the diagnosis. Equally concerning is the absence of disclosed ethical approvals. The dataset does not make clear where the slides originated, nor what procedures were followed to ensure appropriate anonymization practices or ethical review occurred prior to releasing such clinically valuable material into the public domain. This lack of documentation raises questions about both compliance and reproducibility.

## Conclusion

HISTAI represents an important step forward in addressing the scarcity of large, open‐access datasets in computational pathology, and the authors should be commended for making such a resource publicly available. However, the dataset also carries significant downside if used without careful clinical validation.

In short, HISTAI is a valuable resource, but it must be used in the right way. Its limitations highlight the importance of close collaboration between technical researchers and clinicians, ensuring that the dataset's scale and accessibility can be harnessed without compromising scientific rigor or clinical credibility.

## Author contributions statement

JNK and KJH conceived the idea. QZ acquired the data and selected cases. KJH and NGR performed analysis under the supervision of SF and RE. KJH wrote the first article draft and produced figures. All authors reviewed final content and agreed to publication.

## Supporting information


**Figure S1.** Consort chart for updating the diagnosis for adult‐type diffuse glioma (ATDG) cases
**Table S1.** Diagnostic criteria for adult‐type diffuse gliomas

## Data Availability

The HISTAI dataset is available online (https://huggingface.co/histai).
