# Supplementary material for: HISTAI: a valuable dataset with a valuable lesson
Source: J Pathol Clin Res. 2026 Apr 15;12(3):e70089. doi: 10.1002/2056-4538.70089 (PMC13083215; doi:10.1002/2056-4538.70089)
Supplement: Supplementary file 1 — Figure S1. Consort chart for updating the diagnosis for adult‐type diffuse glioma (ATDG) cases Table S1. Diagnostic criteria for adult‐type diffuse gliomas [file CJP2-12-e70089-s001.pdf]

HISTAI: a valuable dataset with a valuable lesson

KJ Hewitt *et al*. *J Pathol Clin Res* https://doi.org/10.1002/2056-4538.70089

Supplementary Figure S1
Supplementary Table S1

Reference numbers refer to the list in the main paper

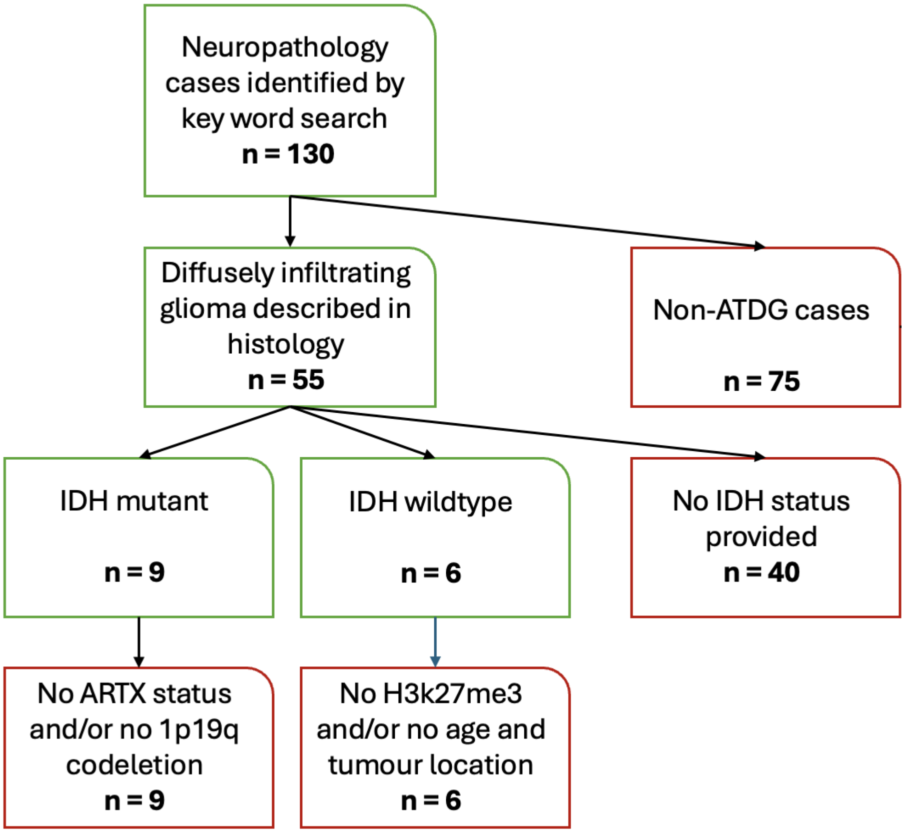

Figure S1. Consort chart for updating the diagnosis for adult-type diffuse glioma (ATDG) cases. We assessed whether the ATDG cases included in the HISTAI dataset met the current diagnostic standards, namely WHO CNS5 [10]. The WHO CNS5 criteria are outlined in Table S1. Boxes with a red border were excluded. No cases met the current diagnostic guidance. No DNA methylation profile information was included in the examined cases.

Table S1. Diagnostic criteria for adult-type diffuse gliomas. In addition to the morphological feature of a diffusely infiltrating glioma, each entity must have the above molecular features, demonstrated by either immunohistochemistry or molecular assay. Alternatively, these entities can be identified via DNA methylation profile analysis; however, no DNA methylation profile information was included in the HISTAI clinical metadata for the cases we examined. Adapted from WHO CNS5 [10].

	Molecular features	Additional notes
Astrocytoma	● *IDH1* codon 132 or *IDH2* codon 172 missense mutation<br>● Loss of nuclear ATRX on IHC, *ATRX* mutation or exclusion of whole arm deletion of 1pand 19q	Demonstration of *IDH* mutation and loss of nuclear ATRX by IHC is sufficient to confirm diagnosis.
Oligodendroglioma	● *IDH1* codon 132 or *IDH2* codon 172 missense mutation<br>● Combined whole-arm deletions of 1p and 19q	Only complete losses of both 1p and 19q chromosome arms are diagnostic for oligodendroglioma. Thus, assays used should be able to detect whole-arm losses.
Glioblastoma	● *IDH*-wildtype<br>● H3-wildtype<br>● One or more of:<br>○ Microvascular proliferation<br>○ Necrosis<br>○ *TERT* promoter mutation<br>○ *EGFR* amplification<br>○ +7/−10 chromosome copy number alterations	Can be diagnosed via absence of IDH1 mutation on IHC in a patient aged ≥55 with histologically classic glioblastoma not located in midline and no history of lower grade glioma.
